# A proof‐of‐principle bite force study using two experimental test denture adhesives and a currently marketed denture adhesive

**DOI:** 10.1002/cre2.256

**Published:** 2020-02-06

**Authors:** Mounir Atassi, Martin R. Ling, Kathy Oneglia, Thomas S. Dilauro

**Affiliations:** ^1^ GSK Consumer Healthcare Weybridge UK; ^2^ TKL Research Bloomfield NJ USA

**Keywords:** edentulism, fixative, prosthesis, taste

## Abstract

**Objectives:**

This proof‐of‐principle, single‐center, randomized, examiner‐blind, crossover study compared two experimental polyvinyl acetate (PVA)‐based denture adhesives (Test Adhesives 1 and 2) with a marketed reference polymethyl vinyl ether/maleic anhydride (PMV/MA)‐based adhesive and no adhesive using incisal bite force area over baseline over 12 hr (AOB_0–12_) in participants with an at least moderately well‐fitting complete maxillary denture. Previous in vitro studies suggested the experimental denture adhesives provided superior performance.

**Materials and Methods:**

Participants were randomized to a treatment sequence such that each received each treatment once. Prior to treatment application (baseline) and at 0.5, 1, 3, 6, 9, and 12 hr following the application, participants bit on a force transducer until their maxillary denture dislodged. Between‐treatment differences in AOB_0–12_ were analyzed using analysis of covariance. For study validity, the reference adhesive was compared with no adhesive. Participants were asked to rate sensory experiences and ease of denture removal.

**Results:**

Twenty‐three participants were included in the modified intent‐to‐treat population. Although Test Adhesives 1 and 2 had a higher mean AOB_0–12_ than no adhesive, differences were not statistically significant. No statistically significant difference was also found between the reference adhesive and no adhesive; hence, study validity was not attained. Participants did not report any clear differences between the test or reference adhesives in terms of taste or feel; however, dentures were easier to remove with the test adhesives versus reference. No treatment‐related adverse events were reported.

**Conclusion:**

Neither the experimental PVA‐based denture adhesives nor the PMV/MA‐based reference product demonstrated a statistically significant difference in incisal bite force AOB_0‐12_ compared with no adhesive. The reasons for these unexpected results is unclear; they suggest that findings of in vitro tests for denture adhesive performance are not always translated to in vivo performance (http://Clinicaltrials.gov: NCT02937870).

## 1 INTRODUCTION

Fully edentulous individuals can be dependent on dental prostheses to carry out the basic oral function of mastication. Retention of complete dentures in the oral cavity is mediated by the interplay of a number of factors, with two of the most important being the establishment of an intimate fit of the intaglio surface of the prosthesis to the underlying tissues and the achievement of an adequate peripheral seal (Felton et al., 2011). Complete denture retention can be negatively affected by changes in hard and soft tissue that comprise the adaptation of the denture surface to the oral tissue, age‐ or medication‐related decline in saliva consistency and volume, and age‐related reductions in bite force and neuromuscular control (Felton et al., 2011).

Although not a substitute for continued denture maintenance to ensure best fit, denture adhesives are widely used to enhance hold and retention of removable dentures, improve mastication, and prevent accumulation of food beneath the denture (Grasso, 2004; Grasso et al., 2000; Marin et al., 2014; Rendell, Gay, Grasso, Baker, & Winston, 2000; Zarb & Felton, 2013). These adhesives are formulated with synthetic hydrophilic polymers that absorb saliva and provide a viscous layer between the oral mucosa of the denture‐bearing tissue and the denture itself (Kumar et al., 2015). One marketed copolymer frequently used in denture adhesive formulations is based on polymethyl vinyl ether/maleic anhydride (PMV/MA) combined with carboxymethyl cellulose (CMC), which hydrates, swells, and becomes sticky on contact with water/saliva.

Polyvinyl acetate (PVA) is an inert and nontoxic polymer that has been used in several denture adhesive formulations; however, these formulations typically use alcohol as a solvent (Guo, Deng, Li, & Prud'homme, 2008). A new water insoluble, pressure‐activated PVA‐based formulation has been developed with a nonalcohol solvent system to try to achieve better full denture hold and food sealing. The novel PVA‐based denture adhesive also contains CMC. This combination of materials delivers a formulation that has been shown in in vitro BioPuls and ISO adhesive strength tests to outperform a marketed PMV/MA‐based denture adhesive, showing greater overall strength and staying stronger for longer (data on file, 2016).

Based on these in vitro results, two experimental adhesive formulations containing different levels of PVA were selected for the current clinical study. The effectiveness of a denture adhesive in improving denture stability can be demonstrated by several methods (Howell & Manly, 1948; Kapur, 1967; Tarbet, Boone, & Schmidt, 1980). Here, the incisal bite force until maxillary denture dislodgement clinical model is used. This has been used previously to determine denture adhesive efficacy between 1 and 12 hr after application and to demonstrate differences between adhesive formulations (Chew, Boone, Swartz, & Philips, 1985; Chew, Phillips, Boone, & Swartz, 1984; Grasso, 2004; Jose et al., 2018; Munoz et al., 2012).

The primary objective of this proof‐of‐principle bite force study was to compare the incisal bite force required to dislodge the maxillary denture over 12 hr with two newly formulated PVA‐based denture adhesives and no adhesive. If statistical significance was not obtained for at least one test adhesive versus no adhesive, then study validity was to be evaluated by comparing the incisal bite force over 12 hr of a marketed PMV/MA‐based reference denture adhesive with no adhesive. Secondary objectives were to compare the 12‐hr incisal bite force between the two test adhesives and between each test adhesive and the reference adhesive. Exploratory objectives included a comparison of incisal bite force at 9 hr for the test adhesives versus the reference adhesive and no adhesive and to determine the oral tolerance of the adhesives. Participants were also asked about the sensory aspects of the denture adhesives and ease of denture removal.

## 2 MATERIALS AND METHODS

This was a proof‐of‐principle, randomized, examiner‐blind, four‐treatment, four‐period, crossover study carried out at a U.S.‐based research facility and registered at http://clinicaltrials.gov (NCT02937870). The protocol was approved by IntegReview Institutional Review Board (registration numbers: IRB00008463, IRB00003657, IRB00004920, IRB00001035, and IRB00006075), and the study was conducted in accordance with the Declaration of Helsinki, the International Conference on Harmonization of Technical Requirements for Registration of Pharmaceuticals for Human Use, and local laws and regulations. One set of minor amendments were made to the protocol that did not affect study flow or outcomes. Anonymized individual participant data and study documents can be requested for further research from http://www.clinicalstudydatarequest.com.

### 2.1 Participants

Potential study participants aged between 18 and 85 years and in good general health were selected from the study center's database. They gave written informed consent prior to the initiation of any study‐related procedures. Participants were required to have a complete edentulous maxillary arch restored with a conventional full acrylic‐based upper complete denture that was at least moderately well‐fitting at the screening visit (Kapur Index, Olshan Modification: retention score ≥2 [fair to excellent], stability score ≥2 [fair to excellent]; Olshan, Ross, Mankodi, & Melita, 1992). If a participant had a partial or full edentulous mandibular arch, this was permitted to have been restored with a stable partial or complete denture or implant‐supported denture. Maxillary and mandibular dentures (if present) were required to be well made based on design and construction criteria, with adequate vertical dimension, freeway space, horizontal occlusal relationships, and border extension; having an acceptable contour and finish; and having acceptable porosity, tissue surfaces, polished surfaces, color, and thickness.

Participants were excluded if they were pregnant or breastfeeding or had any clinically significant or relevant oral abnormality, including oral soft tissue (OST) examination findings, or a serious chronic disease requiring hospitalization or any other condition that could affect study participation; an incisal bite relation that could affect bite force measurements; had severe dry mouth that could affect denture retention; a cardiac pacemaker implant; recently taken/were taking a bisphosphonate or were receiving daily doses of a medication that might interfere with the study; a known/suspected allergy/intolerance to study materials/ingredients; and received an investigational drug within 30 days of screening.

Participants who typically used denture adhesives could continue to use them during washout periods but could not change their routine over the study period. For study visits, participants reported to the clinic without denture adhesive applied to either their maxillary or mandibular denture. To ensure that denture fit was not altered during the study, participants could not have any dental/denture work performed over the study period, unless discussed and permitted by an examiner. Participants could not consume any food or liquid (aside from small sips of water to take medications) for an hour before each treatment visit. During the visit, hot and cold liquid intake was restricted, and standardized meals were provided. Participants were not permitted to chew gum throughout the study period. Smoking, including e‐cigarettes, and the use of chewing tobacco or other tobacco products were prohibited for the duration of screening and on each test day.

### 2.2 Clinical procedures

Participants were required to complete five study visits: screening (Visit 1) and then treatment Visits 2, 3, 4, and 5. Each visit was separated by a washout period of between 1 and 14 days. At the screening visit, potential participants were screened for eligibility and an OST examination was carried out. Participants who met the aforementioned eligibility criteria were required to have a maxillary incisal bite force reading without adhesive of ≤9 lbs (4.1 kg) at the screening visit and prior to treatment application (baseline) at all test visits. At least two of four qualifying bite force measurements at screening needed to be reproducible (±2 lbs [0.9 kg]). To assess incisal bite force, site staff cleaned each participant's denture(s) using Polident^®^ Dentu‐Crème Denture Cleansing Paste (GSK Consumer Healthcare, Brentford, UK) and an Oral B^®^ denture brush (Procter & Gamble, Cincinnati, OH, USA). If a participant had a mandibular denture, this was stabilized prior to insertion with denture adhesive (Super Poligrip^®^ Free Denture Adhesive Cream, GSK Consumer Healthcare, U.S.‐marketed product) applied in two dabs, either side of the interior denture fitting surface, according to product label instructions. The maxillary denture was replaced without adhesive and the examiner recorded triplicate bite force until dislodgement measurements (training bites). A calibrated bite force transducer system was used to measure incisal bite force needed to dislodge the maxillary denture. This system is composed of two plates embedded with a strain gauge that measures denture displacement while biting (Howell & Manly, 1948; Munoz et al., 2012). Following replacement of the maxillary denture (without adhesive), the examiner inserted the bite force plates into the participant's mouth and instructed them to bite until they felt movement on the maxillary denture, at which time the participant released the bite plate. All participants had their bite force assessed by the same examiner to minimize interexaminer variability.

At Visit 2, dentures were cleaned, an OST examination was performed, and if applicable, the lower denture was secured using Super Poligrip^®^ Free Denture Adhesive Cream. The maxillary denture was replaced without adhesive, and three bite force measurements were taken (practice bites), followed by a pretreatment bite force measure (baseline bite). Eligible participants were randomized to order of study product use according to a predetermined schedule generated by the Biostatistics Department of the study sponsor using a Williams Square layout. The four groups were as follows:

- Test Adhesive 1: ingredients: PVA (22.5%), PEG 200 (19.25%), sodium CMC, solvents, emulsifier, and silica;
- Test Adhesive 2: ingredients: PVA (20.0%), PEG 200 (16.75%), sodium CMC, solvents, emulsifier, and silica;
- Reference adhesive (Super Poligrip^®^ Free Denture Adhesive Cream): ingredients: PVM/MA, sodium‐calcium mixed partial salt, CMC, petrolatum, and mineral oil;
- No adhesive.

Study site staff applied a weighed amount (1 ± 0.05 g) of the assigned denture adhesive to each clean dry maxillary denture fit surface as per the application instructions. Test Adhesives 1 and 2 were applied with a precision applicator as one continuous strip along the depth of the maxillary denture ridge (inside the buccolabial and posterior borders), with two smaller strips applied in the middle of the palatal area. The reference adhesive was applied using a precision applicator, according to manufacturer's instructions, as two dabs on either side of the maxillary denture ridge (inside buccolabial and posterior borders) and one dab in the middle of the palatal area.

Clean and dry maxillary dentures were weighed and their masses recorded prior to and immediately following adhesive application for all products. The denture was returned to the participant who rinsed their mouth with water before insertion. In the no adhesive group, the denture was cleaned and dried and then refitted by the participant. Incisal bite force until dislodgement was measured 0.5, 1, 3, 6, 9, and 12 hr after study treatment application and/or denture reinsertion. Only a single application of study adhesive was permitted at each study visit. As stabilization of the lower denture was necessary for bite force measurements to be performed, Super Poligrip^®^ Free Denture Adhesive Cream could be reapplied to the lower denture twice on each test day if the investigator deemed it necessary for bite force measurements. Product packaging was overwrapped to mask identity. To maintain blinding, all application of denture adhesive was performed in an area not accessible by the examiner performing the bite force assessment and participants were instructed not to disclose to the examiner if their dentures had denture adhesive or not.

After the 12‐hr bite force measurement (while the denture was still in place), participants in the dental adhesive groups completed a questionnaire relating to the maxillary denture adhesive only:

- How would you rate your overall opinion of the denture adhesive?
- How much did you like/dislike the taste of the denture adhesive?
- How much did you like/dislike the feel of the denture adhesive?

Responses were 1 (*dislike extremely*), 2 (*dislike moderately*), 3 (*dislike slightly*), 4 (*neither like nor dislike*), 5 (*like slightly*), 6 (*like moderately*), or 7 (*like extremely*). Participants also completed a questionnaire relating to how easy it was to remove the maxillary denture from the mouth, scored as 1 (*not easy at all*), 2 (*slightly easy*), 3 (*moderately easy*), 4 (*very easy*), or 5 (*extremely easy*).

A posttreatment OST was performed before the dentures were cleaned and returned to the participant. All study site staff involved in the collection of bite force and OST examination data were blinded to the distribution and completion of the questionnaires. Visits 3–5 proceeded as for Visit 2.

### 2.3 Safety

Adverse events (AEs) and incidents were collected from the time of the OST examination at screening until 5 days after the last administration of study product. Clinical judgement was exercised by the investigator to assess the relationship between study treatment and occurrence of each AE, with intensity graded as mild, moderate, or severe.

### 2.4 Statistical analysis

Sufficient potential participants were screened so that approximately 21 could be randomized, and at least 18 evaluable participants would complete the study. This sample size was calculated (based on a previous study [Burnett et al., 2018]) to provide approximately 81% power to detect a difference of 2.01 lbs in incisal bite force area over baseline between 0 and 12 hr (AOB_0–12_) for at least one of the test adhesives compared with no adhesive, using two‐sided *t* tests with familywise significance level of 5% based on the Dunnett's adjustment, assuming a residual standard deviation (SD) of 1.929 lbs.

The efficacy analysis was performed on a modified intent‐to‐treat population, defined as all participants who were randomized and had at least one postbaseline assessment of efficacy. The safety population included all participants who were randomized and received study treatment at least once.

The primary efficacy variable was incisal bite force until denture dislodgement AOB_0–12_ for the test adhesives versus no adhesive. The area under the curve (AUC_0–12_) for bite force time was calculated using the trapezoidal method, then AOB_0–12_ was calculated ([AUC_0–12_/12 hr] − baseline bite force). This transformation returned the measurement to the same scale as the original observations while taking in average improvement in bite force AOB by subtracting the baseline value. Linear interpolation was used in the case of missing values. If more than one assessment was missing over the 12‐hr assessment period or if the 12‐hr value or baseline value were missing, AOB was set to missing. Higher values of AOB are indicative of a stronger bite force.

An analysis of covariance (ANCOVA) model was used with AOB values as the response; treatment group, period, and participant‐level baseline as fixed effect factors; period‐level baseline minus participant‐level baseline as covariates; and participant as a random effect. The confidence intervals and *p* values were adjusted using the Dunnett's method so that the overall confidence level for the two confidence intervals was maintained at 95% and the two treatment comparisons were performed with an overall 5% significance level. Success would be achieved if either of the test adhesives was statistically significantly better in terms of AOB_0–12_ than no adhesive. The observed differences for the treatment means were also considered. If statistical significance was not obtained for at least one of the test adhesives versus no adhesive, then study validity was evaluated by comparing the reference adhesive versus no adhesive.

Secondary endpoints were the difference in incisal bite force AOB_0–12_ between the two test adhesives and between each test adhesive and the reference adhesive. Analyses were performed using the AOB calculation and ANCOVA model previously described. Exploratory endpoints included the difference in incisal bite force AOBs over 9 hr (AOB_0–9_) for the test adhesives versus the reference adhesive and versus no adhesive, using the AOB calculation (modified according to time interval) and ANCOVA analysis previously described. The assumptions of normality and homogeneity of variance were investigated for all parametric analyses and considered to be satisfied for both primary and secondary analyses.

Data from the participant‐completed questionnaires were tabulated and presented using descriptive statistics.

## 3 RESULTS

**Figure 1:**
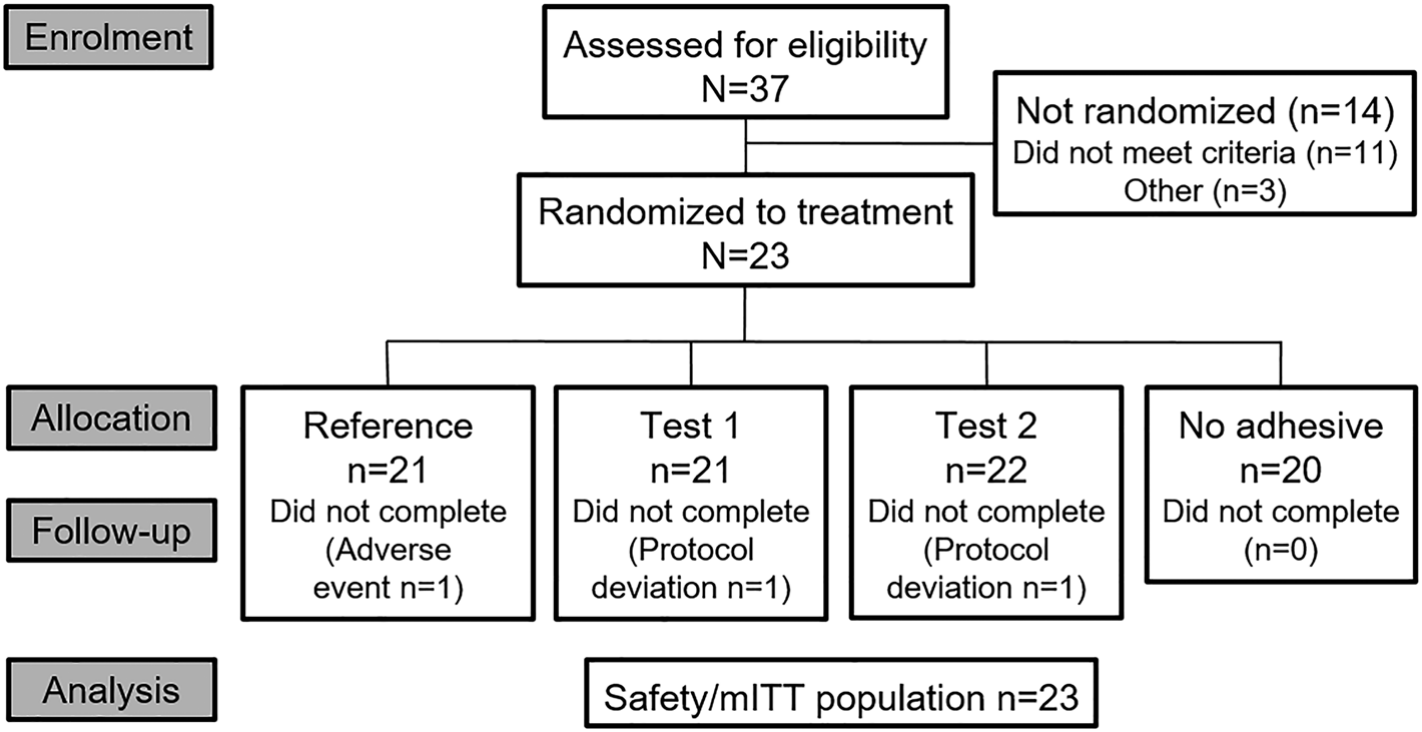
Study flow

The first participant was enrolled in the study in March 2017, with the final participant completing the study in April 2017. A total of 37 participants were screened; 23 were randomized to a treatment sequence and were included in the safety and modified intent‐to‐treat populations (Figure 1). Three participants did not complete the study, one due to an AE and two with protocol deviations.

Of the 23 participants in the safety population, age ranged from 55 to 76 years (mean 64.9 years [*SD* 5.90]), and 18 (78.3%) were female. Participants were Black/African American (*n* = 15; 65.2%), White (*n* = 7; 30.4%) or of multiple races (*n* = 1; 4.3%). The weight of adhesive applied for each denture adhesive treatment was within the tolerance specified in the study protocol (1 ± 0.05 g).

### 3.1 Incisal bite force

#### 3.1.1 Primary efficacy analysis

**Figure 2:**
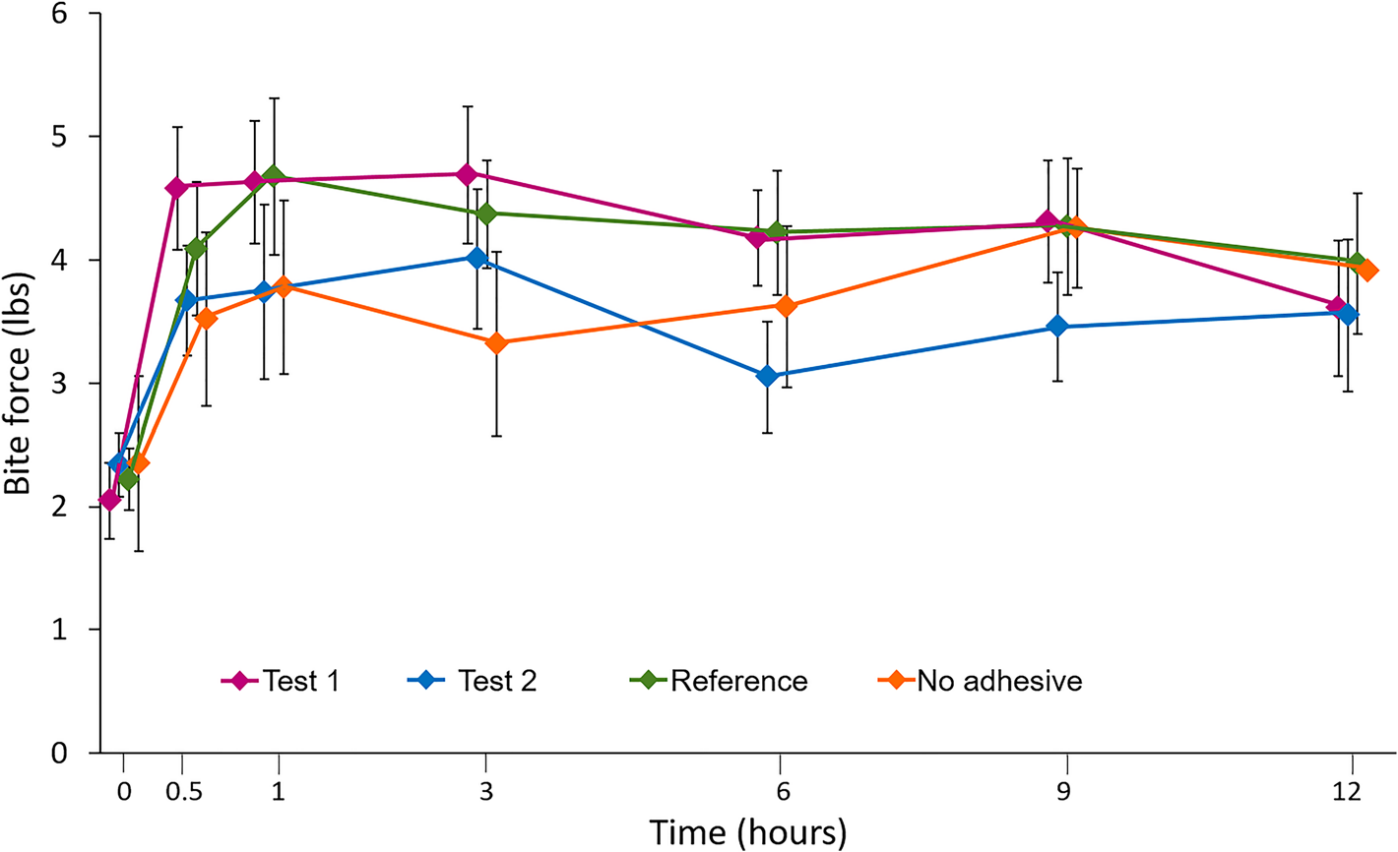
Mean incisal bite force until denture dislodgement over time (modified intent‐to‐treat population)

No statistically significant treatment differences in mean incisal bite force until maxillary denture dislodgement AOB_0–12_ were observed between Test Adhesives 1 or 2 and no adhesive. Likewise, there was no statistically significant treatment differences in AOB_0–12_ between the reference adhesive and no adhesive; hence, study validity was not attained (Table 1; Figure 2).

**Table 1:** Incisal bite force until denture dislodgement area over baseline between 0 and 12 hr (AOB_0–12_) for each study adhesive versus no adhesive (mITT population)

AOB_0–12_ vs no adhesive	Difference^a^, lbs (95% CI)	*p* value
Test Adhesive 1	0.66 [−0.14, 1.4]	.1770
Test Adhesive 2	−0.20 [−0.98, 0.59]	.8321
Reference adhesive	0.59 [−0.20, 1.38]	.1383

Abbreviation: mITT, modified intent to treat.

a Difference is test or reference denture adhesive minus no adhesive, a positive difference favors the first named treatment.

#### 3.1.2 Secondary and exploratory efficacy analyses

As study validity was not attained, only brief details of other study comparisons are provided; full details are in Table S1. For Test Adhesive 1, all between‐treatment differences in incisal bite force AOB_0–0.5_ to AOB_0–9_ were statistically significantly different (*p* < .05), in its favor, compared with no adhesive. There were no statistically significant differences between Test Adhesive 1 and the reference adhesive at any AOB timepoint. For Test Adhesive 2, there were no statistically significant differences between this and no adhesive at any timepoint. Statistically significant between‐treatment differences were observed for incisal bite force AOB_0–9_ and AOB_0–12_ favoring the reference adhesive over Test Adhesive 2 (*p* < .05). There were statistically significant differences (*p* < .05) between Test Adhesives 1 and 2 in favor of the former at AOB_0–6_ to AOB_0–12_. The reference adhesive was associated with a significantly greater incisal bite force than no adhesive for AOB_0–3_ and AOB_0–6_ only (*p* < .05).

### 3.2 Participant questionnaires

#### 3.2.1 Sensory questionnaire

**Figure 3:**
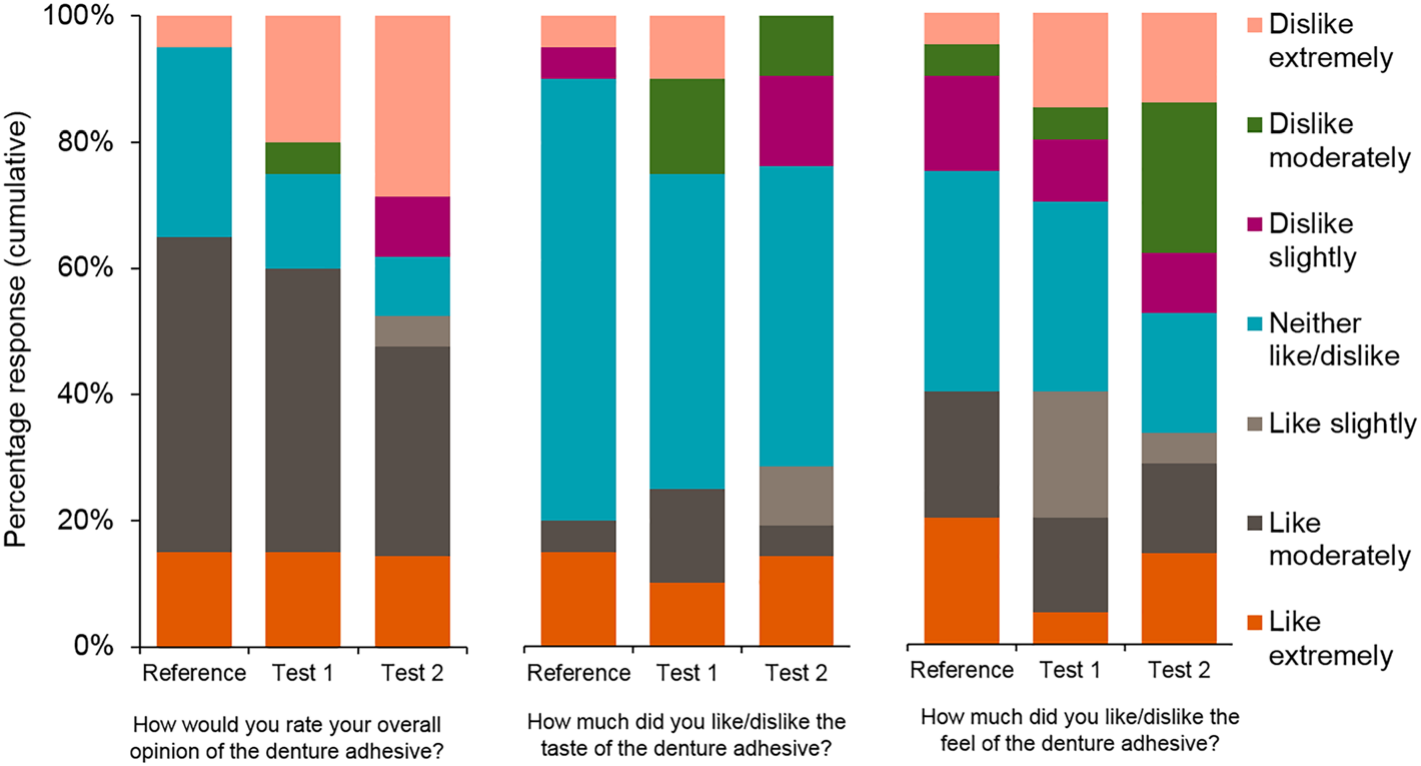
Summary of responses to sensory questionnaire by treatment group (modified intent‐to‐treat population)

Of the participants who completed the questionnaires, 60.0% (12/20) using Test Adhesive 1, 47.6% (10/21) using Test Adhesive 2, and 65.0% (13/20) using the reference adhesive rated their overall opinion as “*liked moderately*” or “*liked extremely*.” Responses demonstrated no clear differences between the test or reference adhesives in terms of taste or feel (Figure 3).

#### 3.2.2 Denture removal questionnaire

The majority of participants reported that it was “*very easy*” or “*extremely easy*” to remove the denture with Test Adhesive 1 (80.0%; 16/20) and Test Adhesive 2 (61.9%; 13/21), and most of those using the reference adhesive reported that it was “*slightly easy*” or “*not at all easy*” to remove the denture (75.0%; 15/20).

### 3.3 Safety

Six treatment‐emergent AEs (TEAEs) were reported in five participants (21.7%), all of which resolved by study completion; none were considered treatment related. One oral TEAE occurred in each adhesive group (mild traumatic ulcer with Test Adhesive 1; mild injury associated with device with Test Adhesive 2; mild gingival ulceration with reference adhesive). Nonoral events occurred in the Test Adhesive 2 group (mild influenza‐like illness) and reference adhesive group (mild abdominal discomfort). One participant from the Test Adhesive 2 group was withdrawn due to device breakage. No serious AEs or incidents were reported.

## 4 DISCUSSION

The primary objective of this proof‐of‐principle study was to investigate denture retention over a 12‐hr period following use of two prototype denture adhesives formulated with a new PVA polymer and modified solvent system for improved denture hold and food sealing. These were compared with a PMV/MA‐based‐marketed denture adhesive and no adhesive treatment.

Neither Test Adhesives 1 or 2 demonstrated statistically significantly greater incisal bite force AOB_0–12_ compared with no adhesive, meaning that the efficacy criterion for success in this study was not met. Furthermore, no statistically significant difference was observed between the marketed reference adhesive and no adhesive for AOB_0–12_. This was unexpected given the results of previous studies of similar design that found that the reference adhesive used here demonstrated statistically significantly greater incisal bite force until dislodgement compared with no adhesive (Jose et al., 2018). In the current study, the mean AOB_0–12_ values were 1.39 with no adhesive and 2.07 with the reference adhesive. In previous studies, mean AOB_0–12_ with no adhesive was slightly higher at around 2.15, whereas mean AOB_0–12_ with the reference adhesive was much higher, at around 4.79 (Burnett et al., 2018; Jose et al., 2018).

Poststudy investigations into study conduct did not reveal any issues that would explain this unexpected result. This study was powered to detect an effect size (mean/*SD*) of 1.042; however, the observed effect size was 0.292, suggesting that the study was potentially underpowered to detect an effect size this small.

A number of candidate formulations were evaluated using in vitro BioPuls and ISO Adhesion tests, both commonly used during development to assess product performance. Both test adhesives selected for this clinical study significantly outperformed the reference adhesive in these models, giving a better overall maximum adhesion strength and maintaining the strength for longer (data on file, 2016). However, in vitro findings were not matched in the clinical setting. This suggests that findings of in vitro tests for denture adhesive performance are not always translated to in vivo performance.

It is of note that application of denture adhesive was different for the reference and test products. No analysis was made to investigate whether our results were influenced by this difference in application styles; therefore, this factor cannot be ruled out. However, further work (data on file) suggests that method of application does not make a significant difference. Study treatments were generally well tolerated. None of the six TEAEs were considered to be related to study treatment, and all had resolved by study completion.

How a product tastes or feels is an important part of any oral product formulation. Using a sensory questionnaire, participants most frequently reported that each adhesive was “*liked moderately*.” No notable preferences for one study adhesive over another were expressed from questions relating to taste or texture. Participants most frequently reported finding it easier to remove their maxillary denture at 12 hr following use of either test adhesive compared with the reference adhesive. The finding that sensory preferences were similar to a marketed formulation means PVA‐based denture adhesives may be an acceptable alternative to PMV/MA‐based adhesives if the formulation is optimized for hold.

## 5 CONCLUSION

In conclusion, there was no statistically significant difference in mean AOB_0–12_ for either test PVA‐based denture adhesives or the marketed reference adhesive compared with no adhesive in participants with an at least moderately well‐fitting complete maxillary denture. The reason for these unexpected results is unclear. As the efficacy validation step for this study was not met, no further conclusions can be made. The study products were generally well tolerated.

## Supporting information

Table S1 Supporting information
