# Supplementary material for: A proof‐of‐principle bite force study using two experimental test denture adhesives and a currently marketed denture adhesive
Source: Clin Exp Dent Res. 2020 Feb 6;6(2):266–73. doi: 10.1002/cre2.256 (PMC7133722; doi:10.1002/cre2.256)
Supplement: Supplementary file 1 — Table S1 Supporting information [file CRE2-6-266-s001.docx]

**Supplementary Table 1.** Between-treatment difference in incisal bite force until denture dislodgement area over baseline (AOB) over different time intervals (modified intent-to-treat population)

|  | **Treatment difference (lbs)* (95% confidence interval) p-value**** | | | | | |
| --- | --- | --- | --- | --- | --- | --- |
|  | **Test adhesive 1 vs no adhesive** | **Test adhesive 1 vs  Reference adhesive** | **Test adhesive 1 vs Test adhesive 2** | **Test adhesive 2 vs no adhesive** | **Test adhesive 2 vs  Reference adhesive** | **Reference adhesive vs  no adhesive** |
| **AOB_0­–12_** | 0.66 (-0.14, 1.47) p=0.1770 | 0.07 (-0.72, 0.87) p=0.8566 | **0.86 (0.06, 1.66) p=0.0352** | -0.20 (-0.98, 0.59) p=0.8321 | **-0.79 (- 1.58, -0.00) p=0.0488** | 0.59 (-0.20, 1.38) p=0.1383 |
| **AOB_0­–9_** | **0.89 (0.05, 1.73) p=0.0378** | 0.12 (-0.72, 0.95) p=0.7811 | **0.96 (0.12, 1.80) p=0.0255** | -0.07 (-0.89, 0.75) p=0.8652 | **-0.84 (-1.67, -0.02) p=0.0446** | 0.77 (-0.05, 1.60) p=0.0660 |
| **AOB_0­–6_** | **1.16 (0.26, 2.06) p=0.0128** | 0.21 (-0.68, 1.09) p=0.6426 | **0.91 (0.01, 1.81) p=0.0479** | 0.25 (-0.63, 1.13) p=0.5759 | -0.70 (-1.58, 0.17) p=0.1112 | **0.95 (0.07, 1.83) p=0.0350** |
| **AOB_0­–3_** | **1.17 (0.30, 2.04) p=0.0092** | 0.22 (-0.64, 1.07) p=0.6159 | 0.82 (-0.05, 1.68) p=0.0638 | 0.36 (-0.50, 1.21) p=0.4065 | -0.60 (-1.44, 0.24) p=0.1601 | **0.96 (0.10, 1.82) p=0.0301** |
| **AOB_0­–1_** | **0.86 (0.11, 1.60) p=0.0247** | 0.26 (-0.48, 0.99) p=0.4866 | 0.69 (-0.05, 1.42) p=0.0672 | 0.17 (-0.56, 0.90) p=0.6410 | -0.43 (-1.15, 0.29) p=0.2364 | 0.60 (-0.13, 1.33) p=0.1071 |
| **AOB_0­–0.5_** | **0.60 (0.09, 1.10) p=0.0216** | 0.26 (-0.24, 0.76) p=0.2958 | 0.46 (-0.05, 0.96) p=0.0738 | 0.14 (-0.35, 0.64) p=0.5685 | -0.19 (-0.68, 0.30) p=0.4342 | 0.33 (-0.17, 0.83) p=0.1856 |

*Difference is first-named treatment minus second-named treatment; a positive difference favors the first named treatment

**P-values in bold indicate statistical significance (p<0.05)
